# Treatment of status epilepticus: Physiology, pharmacology, and future directions

**DOI:** 10.1002/epi4.12725

**Published:** 2023-04-20

**Authors:** Raman Sankar

**Affiliations:** ^1^ Departments of Neurology and Pediatrics David Geffen School of Medicine at the University of California, Los Angeles Los Angeles California USA

**Keywords:** antiseizure medications, extrasynaptic modulation, postsynaptic modulation, presynaptic modulation, status epilepticus

## Abstract

The review presents retrospective, present views and future perspectives on the treatment of status epilepticus (SE). First, presynaptic, postsynaptic, and extrasynaptic mechanisms underlying sustaining ongoing seizure activity are highlighted. Next, mechanism‐based choices of antiseizure medications capable of promptly arresting SE are introduced. Finally, challenges associated with translating the advances in laboratory research in clinical practice are discussed.

**Key Point**

- Status epilepticus (SE) becomes refractory to antiseizure medications (ASM) due to progressive pre‐ and postsynaptic perturbations
- Important postsynaptic changes involve the trafficking of receptors for GABA and glutamate
- Selective Na^+^ channel inhibitors counteracting excitation without affecting fast‐spiking GABAergic interneurons may be promising in treating SE
- Efficacy of GABAergic inhibition may be augmented by ASMs targeting extrasynaptic GABA receptors and by activators of the KCC2 transporter

## 1 INTRODUCTION

At the recently held 8th London‐Innsbruck Colloquium on Status Epilepticus and Acute Seizures, held in Salzburg September 17–20, 2022, Professor Claude Wasterlain was honored as the keynote lecturer on the topic of “50 years of Status Epilepticus Research.” This article is dedicated to Professor Wasterlain in appreciation of his many contributions to our understanding of the pathophysiology of status epilepticus (SE) and the pharmacological underpinnings of its present treatment, and what newer therapeutic options may emerge in the near future.

## 2 PRESYNAPTIC MECHANISMS IN SUSTAINING ONGOING SEIZURE ACTIVITY

The maintenance of ictal activity during SE requires continued release of excitatory neurotransmitter from presynaptic sites. As recently reviewed by Xue et al,^1^ exocytosis of neurotransmitters is a Ca^2+^‐regulated process that requires the participation of Ca^2+^ sensors. The two important Ca^2+^ sensors include calmodulin and synaptotagmin. Calmodulin appears to be involved in the earlier exocytotic steps prior to fusion, such as vesicle trafficking, docking, and priming by acting as a high‐affinity Ca^2+^ sensor activated at sub micromolar level of Ca^2+^, while synaptotagmin is involved in triggering the final stages of membrane fusion (synaptic vesicle to presynaptic membrane) due to its low‐affinity binding triggered by micromolar boosts in Ca^2+^ levels. Wasterlain and associates demonstrated that during SE membrane calmodulin kinase II was translocated to the cytosol and phosphorylated and that this process was no longer coupled to the entry of sodium.^2^ Such a phenomenon would explain how sodium channel‐blocking antiseizure medications (ASM) such as phenytoin may fail to control ongoing seizure activity.

## 3 POSTSYNAPTIC MECHANISMS (RECEPTOR TRAFFICKING) IN SUSTAINING ONGOING SEIZURE ACTIVITY

In subsequent years, the Wasterlain laboratory^3^ and the laboratory of Jaideep Kapur^4^ showed that there were important postsynaptic changes that rendered SE to endure even in the face of treatment with barbiturates or benzodiazepines. Both laboratories independently demonstrated a progressive loss of gamma‐aminobutyric acid (GABA) GABA_A_ receptors due to endocytosis of these receptors triggered by ongoing seizure activity.^3^, ^4^ Further insight into this process came from the Moss laboratory (Terunuma et al)^5^; they identified a SE‐induced deficit in phosphorylation of a serine residue in the β3 subunit by protein kinase C isoforms that facilitated endocytosis of the GABA_A_ receptors. The authors suggested that enhancing the phosphorylation or blocking the interaction of the binding of the dephosphorylated β3 subunit to the clathrin adaptor may represent a novel approach to ameliorate SE. The Goodkin laboratory (Joshi et al)^6^ focused on the activity‐dependent trafficking of γ2 subunit's role in pharmacoresistance and demonstrated that the treatment of SE‐treated slices with protein phosphatase inhibitors FK506 or okadaic acid restored the surface expression of the γ2 subunit‐containing GABA_A_ receptors and the mIPSC amplitude. Kapur and Macdonald^7^ had reported several years earlier on a loss of responsiveness to benzodiazepine treatment in a rodent model of status epilepticus and had demonstrated this phenomenon to be associated with a reduction in benzodiazepine and Zn^2+^ sensitivity of hippocampal granule cells.

These findings involving GABA receptor trafficking in the laboratory have strong clinical correlates. A study of status epilepticus patients at San Francisco General Hospital by Lowenstein and Alldredge^8^ found that 80% of those patients who were treated within 30 minutes of the onset of seizures responded well to first‐line treatment while over 60% of the patients who were in SE for two or more hours before initiation of therapy failed to respond to first‐line medical treatment. Further clinical data consistent with the evolving changes in the brain with ongoing seizure activity was reported by Alldredge et al^9^ in a study of the effect of prehospital diazepam therapy on pediatric SE patients treated at San Francisco General Hospital. They found that prehospital diazepam therapy was associated with SE of shorter duration (32 minutes vs 60 minutes; *P* = 0.007) and a reduced likelihood of recurrent seizures in the emergency department (58% vs 85%; *P* = 0.045).^7^

The laboratory of Jaideep Kapur has also investigated aspects of α‐amino‐3‐hydroxy‐5‐methyl‐4‐isoxazolepropionic acid (AMPA) receptor trafficking.^10^, ^11^ They demonstrated that GluA2 subunits of AMPA receptors were reduced in the hippocampus during refractory SE, rendering those receptors more Ca^+2^ permeant.^10^ Subsequent studies in that laboratory by Joshi et al found an increase in the surface expression of GluA1 subunits during SE.^11^ Treatment with MK‐801 prevented the increased surface expression of the GluA1 subunits. In this study involving high‐dose pilocarpine‐treated animals, MK‐801 alone did not significantly decrease the EEG power, but the combination of MK‐801 and diazepam did.

Mazarati and colleagues described a model of self‐sustaining SE (SSSE) after brief electrical stimulation of the perforant path^12^, which proved to be suitable for the study of the time‐dependent decrease in the effectiveness of ASM during SSSE and to explore other approaches than sodium channel antagonists and GABA_A_ receptor allosteric modulators.^13^ Both diazepam and phenytoin in appropriate doses prevented the establishment of SSSE when administered 10 minutes prior to perforant path stimulation (PPS), but diazepam was significantly less effective in attenuating SSSE if given 10 minutes after the end of 30 minutes of PPS. Likewise, phenytoin aborted SSSE when administered 10 minutes after 30 minutes of PPS but showed vastly decreased efficacy 40 minutes after 30 minute PPS.^13^ Interestingly, intrahippocampal injection of the AMPA/kainate receptor blocker 6‐cyano7‐nitroquinixaline‐3‐dione (CNQX, 10 nmol) transiently suppressed seizures, which reappeared 4–5 hours later. Blockade of NMDA receptor at the PCP site by MK‐801 (0.5 mg/kg, i.p.) or ketamine (10 mg/kg, i.p.), as well as at the glycine allosteric site by intrahippocampal 5,7‐dichlorokynurenic acid (5,7‐DCK, 10 nmol), rapidly and irreversibly aborted both behavioral and electrographic manifestation of seizure activity.^14^ The authors concluded that the maintenance phase of SSSE depended on the activation of NMDA receptors. As mentioned above, the surface expression of Ca^+2^ permeant AMPA receptors appears to be sensitive to treatment with MK‐801.^11^

Subsequent research in the Wasterlain laboratory described the progressive accumulation of N‐methyl‐D‐aspartate (NMDA) receptors (immunocytochemistry signal was for the NR1 subunit) on the surface of the soma and dendrites of CA3 pyramidal cells subjected to lithium‐pilocarpine SE; electrophysiologic confirmation was obtained by the increase in NMDA‐mEPSC amplitude.^15^ The direct observation of increasing NMDAR function, along with the previously described role of NMDAR in the mobilization of AMPAR (GluA1 subunits) to the cell surface would suggest that an NMDAR antagonist would be a logical choice early in the course of SE.

Clinical experience with the use of an NMDAR antagonist, using an approved medication, ketamine, exemplifies the typical problems in translation in the management of SE. A large number of reports are isolated case studies, and the deployment of ketamine therapy was rather late in the course of SE. In a retrospective multicenter study,^16^ involving 10 academic medical centers in North America and Europe (58 subjects), ketamine was introduced after a median of 9 days of SE as part of a multidrug regimen that ranged from 2 to 12 concurrent medications. While acknowledging that the etiology of SE in these cases ranged from anoxia to other unknown causes of acute brain injury rather than SE induced by experimental conditions in rodents, we note that the timing of deployment of ketamine therapy is likely well past the optimum period for a favorable response. We also posit that regardless of the precipitating cause of SE, at some point, ongoing and uncontrolled seizure activity itself becomes a proximal contributor to the situation resembling SSSE. An earlier report^17^ involving 9 pediatric patients with epilepsy in refractory SE had initiated ketamine therapy after a median of 6 days and after conventional anesthetics; despite that delayed start, 6/9 responded with the resolution of seizures, while 2 required surgical removal of epileptogenic focal cortical dysplasia. These experiences suggest that the adoption of ketamine therapy much earlier in the course of SE may indeed prove to be valuable in clinical practice.

## 4 OTHER PHARMACOLOGICAL TARGETS FOR THE TREATMENT OF SE AND COMBINATION THERAPIES

Mazarati and Wasterlain^18^ studied the anticonvulsant actions of neuropeptides in the previously alluded SSSE model. The neuropeptides were injected directly into the hilus of the dentate gyrus. While somatostatin and neuropeptide Y provided for transient suppression of spikes and seizures, an irreversible suppression of seizures was achieved by dynorphin and galanin. The anticonvulsant potential of galanin has been extensively studied and reviewed by Mazarati et al.^19^ Synthetic galanin analogs that are brain‐permeant have been synthesized^20^ and the most active one, NAX 5055, has been tested in epilepsy models,^21^ but its efficacy in SE has not been published.

The noncompetitive AMPA receptor antagonist perampanel was shown to be effective when lithium‐pilocarpine‐induced SE in rats became refractory to diazepam,^22^ but it is not clear whether seizures recurred, as was observed in the SSSE model induced by PPS when the AMPA antagonist CNQX was employed.^14^ Limitations to clinical use include the lack of availability of a parenteral form and the long time required to achieve an adequate steady‐state concentration. In a small clinical series from a neurological intensive care unit, when perampanel was given after a median of four ASM, perampanel could only ameliorate seizure activity in a few patients with refractory SE.^23^ The authors speculated that the modest results may in part be due to the long duration of SE before the administration of perampanel, and the conservative dosing. The translational gulf between preclinical experiments and clinical trials thus far remains characterized by the extraordinarily long duration of SE before novel therapies have been employed. We remarked on this earlier in our discussion of clinical experience with ketamine.^16^

The potential value of early combinatorial therapy (polypharmacy) in treating SE has been explored. Löscher applied this concept to rats undergoing lithium‐pilocarpine‐induced SE to treat the acute condition and the long‐term consequences.^24^ This study involved a cocktail containing scopolamine, phenobarbital, and diazepam; the possible benefit of scopolamine may be specific to cholinergic‐stimulated SE. The use of intravenous diazepam followed by intravenous loading with phenobarbital or phenytoin has long been a common clinical practice.

Based on the concepts alluded to earlier regarding the trafficking of synaptic GABA receptors^3^, ^4^ and AMPA receptors,^10^, ^11^ the latter dependent on NMDAR activation, the Wasterlain laboratory deployed polytherapy using combinations of allosteric GABA_A_R agonists midazolam or diazepam with an NMDAR antagonist, dizocilpine or ketamine.^25^, ^26^, ^27^ The combinations terminated high‐dose diazepam‐refractory SE, reduced neuronal damage, and limited spatial memory deficits, and the occurrence of spontaneous recurrent seizures (SRS). The superior performance of the combination of diazepam and ketamine to either one of those compounds alone in terminating SE produced by cholinergic stimulation was also observed by Martin and Kapur.^28^ The Wasterlain laboratory also demonstrated that triple therapy involving midazolam, ketamine, and valproate was especially effective in terminating refractory SE in rats (induced by lithium‐pilocarpine treatment); the combination was superior to triple‐dose of any of those agents alone or sequential therapy involving those three agents.^29^ The authors drew a comparison with the clinical practice of sequential use of medications in the treatment of SE and remarked that early correction of maladaptive receptor trafficking with combination therapy needs clinical evaluations. They also noted that the importance of dealing with receptor trafficking was highlighted by the superiority of midazolam‐ketamine‐valproate therapy to midazolam‐fosphenytoin‐valproate therapy.

## 5 TRAFFICKING DIFFERENCE BETWEEN POSTSYNAPTIC AND EXTRASYNAPTIC GABAR PRESENTS NEW OPPORTUNITIES

The discovery of GABA_A_ receptors containing δ subunits in extrasynaptic locations, which do not bind benzodiazepines and possess α4, α5, or α6 subunits to produce a sustained hyperpolarizing current (also called tonic current) upon activation as distinct from the synaptic receptors containing α1, α2, or α3 subunits that mediate a transient current (rapidly inactivated, also called phasic), contain a γ subunit that facilitates benzodiazepine binding to provide for positive allosteric augmentation of that current, has opened novel pharmacologic tools to approach the challenge of refractory SE.^30^, ^31^, ^32^ The extrasynaptic receptors are highly sensitive to low concentrations of GABA and activation can be enhanced by neurosteroids like allopregnanolone and tetrahydrodeoxycorticosterone (THDOC). The synaptic receptors contain β2 or β3 subunits, while the extrasynaptic receptors contain β1 subunits.^31^ As mentioned earlier, SE selectively decreases the phosphorylation of GABA_A_Rs on serine residues 408/9 (S408/9) in the β3 subunit by the intimately associated protein kinase C isoforms.^5^ The unphosphorylated S408/9 represents a patch‐binding motif for the clathrin adapter AP2 that promotes endocytosis. This would suggest that the extrasynaptic GABA_A_ receptors (lacking β3 subunits) might not get internalized due to seizure activity. Mangan et al^33^ had studied cultured hippocampal pyramidal neurons and had identified two kinds of GABA_A_R with distinct subunit stoichiometry representing those present in the synapses versus those in extrasynaptic loc Indeed, in hippocampal slices obtained from animals undergoing SE, Goodkin et al^34^ found that the surface expression of the GABAR β2/3 and γ2 subunits was reduced, whereas that of the δ subunit was not. Electrophysiological recordings from dentate granule cells in SE‐treated slices demonstrated a reduction in GABAR‐mediated synaptic inhibition but not tonic inhibition. These observations point to the potential for modulators of tonic GABAergic inhibition such as neurosteroids, in the treatment of SE. It should also be acknowledged that refractory SE has been treated with general anesthetics, which also mediate tonic GABAergic inhibition. However, their other activities (not discussed in this review) render them a risky treatment modality with high associated mortality.

## 6 ALLOPREGNANOLONE AND GANAXOLONE

Allopregnanolone ((5a‐pregnan‐3a‐ol‐20‐one) is an endogenous metabolite of progesterone, with poor oral bioavailability while ganaxolone (GNX), its 3β‐methyl synthetic analog possesses oral bioavailability and metabolic stability. The anticonvulsant properties of GNX were initially described in 1993.^35^ The ability of allopregnanolone and GNX to stop diazepam‐refractory SE in rats were described by the laboratory of Rogawski.^36^, ^37^ An intravenous preparation of GNX was used by Saporito et al^38^ to block lithium‐pilocarpine‐induced, diazepam‐resistant SE. These neurosteroids are active at both synaptic and extrasynaptic GABAR, but the extrasynaptic receptors are not progressively lost during the course of SE due to endocytosis.

Pediatric case reports of super‐refractory SE‐treated with allopregnanolone first appeared in 2014.^39^ In this report, patient one was started on allopregnanolone only by day 52 under emergency authorization by the FDA, and patient two received it by day 15. Two adult cases of allopregnanolone to treat super‐refractory SE were subsequently published, curiously entitled as first‐in‐man allopregnanolone use, in 2017^40^! Again, allopregnanolone therapy had been initiated after multiple failures to wean the patient off pentobarbital coma. Likewise, pediatric case reports describing the administration of GNX to treat super‐refractory pediatric SE have been described^41^ and in both cases, the implementation followed the failure of pentobarbital coma.

## 7 TIME IS BRAIN IN THE TREATMENT OF SE

### 7.1 Timely addressing of GABAR trafficking

Treatments designed to overcome adverse receptor trafficking during SE must be employed much earlier than the clinical transition of refractory SE to super‐refractory SE—for the purpose of this review, we have no interest in rehashing the definitions of super‐refractory SE, since our point of view is to emphasize the implementation of physiologically and pharmacologically appropriate treatment at a fairly early course of SE. There is no scientific basis to initiate anesthetic coma, wait for 24 hours or more, and delay the deployment of medications that may influence adverse receptor trafficking effectively.

Finally, the report of an open‐label, dose‐finding, phase 2 trial of GNX has been published^42^ in which patients received this study medication after the failure of 2nd line treatment and *before proceeding to intravenous anesthesia*. Sixteen of 17 patients (94%) achieved and maintained SE cessation for 14 hours following GNX initiation. The median time to SE cessation following initiation of GNX infusion was 5 minutes. Of these patients, 15 (94%) achieved SE cessation within 30 minutes of initiating GNX, and one (6%) at ~4 hours postinitiation.

The importance of the timing of initiation of GNX is highlighted by a failed trial of allopregnanolone^43^ in which administration of allopregnanolone (SAGE‐547) followed, in most cases, after 24 hours or more on intravenous anesthesia. This author's contention is that new regimens for treating SE should appear much earlier than when the situation has progressed to super‐refractory SE. The clinical definition of super‐refractory SE may represent a biological point of no return for many patients.

The findings from the GNX phase 2 study^42^ have encouraged the design of a randomized, placebo‐controlled study of GNX^44^ in which the investigational product will be added to the standard‐of‐care regimen before IV anesthetics during the treatment of SE. In the randomized, placebo‐controlled study^44^ GNX infusion may begin after a benzodiazepine and two or more second‐line intravenous ASM from a list. The GNX will be initiated with a bolus followed by a continuous infusion for 36 hours, followed by a 12 hours taper. The primary outcome measures are (1) the proportion of participants with status epilepticus cessation within 30 minutes of IP (investigational product, i.e., GNX) initiation without medications for the acute treatment of status epilepticus. SE cessation is determined by clinical and EEG findings; (2) the proportion of participants with no progression to IV anesthesia for 36 hours following IP initiation. The secondary outcome measures are (1) no progression to IV anesthesia for 72 hours following IP initiation and (2) time to SE cessation following IP initiation. Further details can be found at the clinicaltrials.gov website.^44^

### 7.2 Timely addressing of AMPAR trafficking

Another proposal to leverage the potential efficacy and neuroprotective possibility^45^ of early use is being made by Kapur and associates (Coles et al).^46^ In their proposal, two ketamine doses, 1 and 3 mg/kg, each infused over 5 minutes will be added to the second‐line ASM in the treatment of SE. In their article, they review comprehensively the animal data pertaining to the use of ketamine, as well as the human safety data, emphasizing emergency department experience with this medication.^46^

## 8 THE FUTURE—NEW POSSIBILITIES ON THE SODIUM CHANNEL FRONT

When we discuss ASMs that mediate their action by use‐dependent sodium channel blockade, we usually do not differentiate between the fast‐inactivating Na‐current (transient, I_NaT_), associated with action‐potential (AP) generation and the noninactivating or persistent Na‐current (I_NaP_); the latter which may resemble a paroxysmal depolarization shift (PDS), historically attributed to being calcium‐mediated. I_NaP_ contributes to depolarizing plateau potentials, on which a train of APs can occur, corresponding to an EEG spike on the scalp when it involves a sufficient number of neurons. Eukaryotic Na_v_ channels are composed of one α subunit, which can be coupled to one or two β subunits. The different α subunits define the distinct Na_v_ channel subtypes and contain the receptor sites for drugs and toxins that act on Na_v_ channels.^47^ Many epilepsies have an underlying mutation in genes coding for the α subunit. Important to our discussion are the two following simplifications: (1) Na_v_1.6, encoded by SCN8A, can augment I_NaP_—this is present only in excitatory neurons (2) Na_v_1.1, encoded by SCN1A gives rise to I_NaT_ supporting AP generation in excitatory neurons and inhibitory GABAergic interneurons.^48^ Further, Na_v_1.2 is also mainly present in excitatory neurons and is encoded by SCN2A. All the above‐mentioned currents can be seen in excitatory cells, but Na_v_1.1 is very important in inhibitory interneurons, and the APs generated therein result in GABA release. Hence, the classic advice is that traditional (nonselective) Na^+^ channel‐blocking ASMs should not be used in Dravet syndrome, the most common etiology of which is impairment in Na_v_1.1 resulting in impaired inhibition. Nonselective Na^+^ channel inhibitors would exacerbate the preexisting problem with interneuron function by blocking Na_V_1.1.

Most presently available ASMs with Na^+^ channel blockade do not differentiate between I_NaT_ and I_NaP_. However, the new ASM cenobamate marks a radical departure in this regard. Cenobamate had little effect on the peak component of transient Na^+^ current (I_NaT_) induced by brief depolarizing step pulses, but it potently inhibited the noninactivating persistent component of I_Na_ (I_NaP_).^49^ This suggests that cenobamate can *modify excitability in principal neurons without compromising inhibitory interneurons*. Indeed, experience in deploying cenobamate in medication‐resistant patients diagnosed with Dravet syndrome is growing in our clinics, and a recent report^50^ documents 4 adult patients with severe, drug‐resistant Dravet patients exhibiting more than 80% reduction in seizures with a follow‐up up to 542 days. Its potential use in SCN8A gain‐of‐function disorders is also obvious.

Beyond the I_NaT_ and the I_NaP_, certain neurons also exhibit what has been called the “resurgent sodium current (I_NaR_)” that activates upon membrane repolarizations.^51^ It is believed to result from an atypical path of Na^+^‐channel recovery from inactivation. Castelli et al showed that the parahippocampal region and part of the hippocampal formation are sites of major I_NaR_ expression.^52^ The I_NaR_ is predominantly transmitted through Na_v_1.6 mechanisms and contributes to hyperexcitability.

In addition to limiting I_NaP_, cenobamate also acts as a positive allosteric modulator of high‐affinity GABA_A_ receptors, activated by GABA at a site independent of the benzodiazepine binding site and efficiently enhances tonic inhibition in hippocampal neurons.^53^ Thus, cenobamate can at once selectively target principal cells, preserving the full inhibitory potential of interneurons, and also contribute to the inhibition of principal cells at the extrasynaptic GABA receptors, which resist internalization during the course of SE. Thus, this combination of mechanisms could be a powerful approach to early intervention in SE. At present time, no intravenous formulation of cenobamate is available.

Medicinal chemists at Xenon Pharmaceuticals have reported on a series of aryl sulfonamides as CNS‐penetrant, isoform‐selective Na_V_1.6 inhibitors, which also displayed a potent block of Na_V_1.2.^54^ Optimization of compound structural features focused on increasing selectivity over Na_V_1.1, improving metabolic stability, and reducing active efflux from the brain (multiparameter optimization). Compound XPC‐6444 exhibited a Na_V_1.6 IC_50_ of 0.041 μM, while the IC_50_ for Na_V_1.1 was >10 μM (and a respectable 0.125 μM for Na_v_1.2). It was active in the DC maximal electroshock model, 6 Hz‐induced seizure model, as well as SCN8A gain‐of‐function mice carrying a mutation identified in a human EIEE13 patient. XEN901 is a compound from Xenon since licensed to Neurocrine Biosciences (NBI‐921352), and is a Na_V_1.6 selective inhibitor that affords potent stabilization of inactivation, inhibiting Na_V_1.6 currents, including resurgent and persistent Na_V_1.6 currents.^55^ The isoform‐selective profile of NBI‐921352 led to a robust inhibition of action‐potential firing in glutamatergic excitatory pyramidal neurons, while sparing fast‐spiking inhibitory interneurons, where Na_V_1.1 predominates.

## 9 K^+^/CL^−^ COTRANSPORTER 2 (KCC2) AS A POTENTIAL TARGET

In a low Mg^2+^ model of SE in slices, diazepam lost its antiseizure efficacy and conversely exacerbates epileptiform activity, as did phenobarbital.^56^ The authors found that persistent SE‐like activity was associated with a reduction in GABA_A_ receptor conductance and Cl^−^ extrusion capability. This observation opens the possibility that enhancing the function of KCC2 transporter responsible for chloride extrusion could contribute to gaining pharmacosensitivity of the SE to benzodiazepines and barbiturates. The Moss laboratory found that inhibition of a with‐no‐lysine (WNK) kinase by WNK463 resulted in enhanced KCC2‐mediated Cl^−^ extrusion.^57^ WNK463 was effective in vivo in C57BL/6 mice that underwent kainic acid‐induced SE by preventing diazepam resistance. A small molecule KCC2 activator, OV350, is under development by Ovid Therapeutics—no published data are available at this time.

## 10 CONCLUDING REMARKS

Little has changed in the clinical management of SE during the three decades plus of this author's experience, even as basic science knowledge has accumulated pointing at new approaches. Clinical adoption of general anesthesia is readily adopted by default in the management of refractory SE, despite the high mortality with barbiturate anesthesia and the risks associated with propofol.^58^ Animal studies document the adverse cardiac effects of pentobarbital and pentothal to be attributable to impairment of oxidative phosphorylation.^59^ In this setting, managing hypotension with pressors and inotropic agents only magnifies the cardiac energy imbalance, leading to failure.

Thus, we are encouraged to see the new GNX trial, where the test drug will be added to second‐line therapy ahead of intravenous anesthesia. The proposal to add ketamine to second‐line therapy is also in the right direction. Finally, the potential for Na_v_1.6 selective agent that avoids impairing GABAergic interneuron function by blocking Na_V_1.1 is tantalizing—we await the availability of compounds suitable for intravenous administration and there is a reason for optimism.

## CONFLICTS OF INTEREST STATEMENT

Dr. Sankar is a speaker for SK Life Science, a manufacturer, and a marketer of cenobamate, which is covered in this article. There are no other conflicts of interest to disclose.

## ETHICAL APPROVAL

I confirm that I have read the Journal's position on issues involved in ethical publication and affirm that this report is consistent with those guidelines.
